# Assessing acceptability of pre-exposure prophylaxis (PrEP) among participants in an HIV vaccine preparedness study in southwestern Uganda

**DOI:** 10.1371/journal.pone.0271104

**Published:** 2022-07-29

**Authors:** Sarah Nakamanya, Rachel Kawuma, Denis Kibuuka, Sylvia Kusemererwa, Sheena McCormack, Eugene Ruzagira, Janet Seeley, on behalf of the PrEPVacc Study Group

**Affiliations:** 1 Medical Research Council/Uganda Virus Research Institute (MRC/UVRI) and London School of Hygiene and Tropical Medicine (LSHTM) Uganda Research Unit, Entebbe, Uganda; 2 MRC Clinical Trials Unit at UCL, London, United Kingdom; 3 London School of Hygiene and Tropical Medicine, London, United Kingdom; HIV/STI Surveillance Research Center and WHO Collaborating Center for HIV Surveillance, Institute for Future Studies in Health, Kerman University of Medical Sciences, ISLAMIC REPUBLIC OF IRAN

## Abstract

**Background:**

Daily oral pre-exposure prophylaxis (PrEP) use is highly effective against HIV infection. However, the uptake of PrEP among individuals at high-risk of HIV acquisition in sub-Saharan Africa varies because of availability and acceptability. We assessed the acceptability of PrEP among participants in a prospective HIV vaccine preparedness study in Masaka, southwestern Uganda.

**Methods:**

From November 2018 to August 2019, 20 participants (10 female) were purposively selected for in-depth interviews (IDIs) at 3 and 9 months’ post-enrolment in the vaccine preparedness study. Four focus group discussions (FGD) (two among men) were conducted with 29 individuals categorized as: younger (18–24 years) men, younger (18–24 years) women, older (≥30 years) men, and older (≥30 years) women. Apart from IDI specific questions on recent life history including work experience, relationship history and places lived, topics for IDIs and FGDs included knowledge of HIV, perceptions of HIV risk (including own risk), knowledge of and use of PrEP. The Theoretical Framework of Acceptability was used to structure a thematic framework approach for data analysis.

**Results:**

Participants understood that PrEP was an oral pill taken daily by HIV negative individuals to prevent acquisition of HIV. Overall, interest in and acceptability of PrEP was high, more than half expressed positivity towards PrEP but were not ready to initiate taking it citing the burden of daily oral pill taking, related side effects, stigma and distrust of PrEP. Fourteen participants (from IDI and FGD) initiated PrEP, although some (one FGD and two IDI participants) stopped taking it due to side effects or perceived reduced risk.

**Conclusion:**

We observed a keen interest in PrEP initiation among our study participants. However, a limited understanding of PrEP and associated concerns impeded uptake and sustained use. Hence, interventions are needed to address end-user challenges to increase uptake and support adherence.

## Introduction

The HIV-epidemic continues to be a major global public health problem with around 1.5 million new infections in 2020, of which over two-thirds were in sub-Saharan Africa (SSA) [1]. Almost one-half of new global HIV infections occur among key populations, such as men who have sex with men (MSM), people who inject drugs, sex workers, transgender people and their sexual partners, migrants, truck drivers, fisher-folk, adolescent girls and young women, prisoners, plantation workers, miners, ‘boda-boda’ (motorcycle taxi) riders and uniformed services personnel [2, 3]. In SSA, 39% of new HIV infections in 2020 were among key populations [1]. Data reported to UNAIDS in 2019 on uptake of combination HIV prevention services revealed that among key populations, less than 10% had access to multiple prevention services in parts of Africa, including Uganda [4].

Oral HIV pre-exposure prophylaxis (PrEP), the use of antiretroviral drugs (ARVs) by HIV-negative people to reduce the risk of HIV acquisition, is a highly effective HIV prevention tool and has the potential to substantially reduce HIV incidence among members of key populations [5]. PrEP is recommended by WHO and implemented in an increasing number of countries in SSA [6, 7]. Although willingness to use PrEP appears to be high among certain populations in SSA, uptake rates are low [8, 9].

The acceptability and interest in an intervention such as PrEP is key to its success as individuals are more likely to take it up and/or adhere to it if they consider it acceptable and beneficial to them [10, 11]. Factors that influence individuals’ acceptability of an intervention have been shown to include its appropriateness in addressing the problem, and suitability for an individual’s lifestyle, convenience, social acceptability and effectiveness [12]. These factors could also determine adherence to that intervention.

In the current study, we assessed the acceptability of PrEP among participants in a prospective HIV vaccine preparedness study in Masaka, southwestern Uganda. All participants received information and counselling about risk reduction strategies and services, including PrEP, and referral to services if desired.

### Theoretical framework

We adopted the Theoretical Framework of Acceptability (TFA) [13] to assess PrEP acceptability. Data were analysed in relation to the seven components of the TFA. For example, we looked at individual’s feelings about taking up PrEP (affective attitude), their perceived/experienced amount of effort required to take PrEP (burden), the extent to which PrEP suited their lifestyles (ethicality), extent to which participants understood PrEP and how it works (intervention coherence), the extent to which benefits/profits/values were/must be given up to take PrEP (opportunity costs), the extent to which PrEP was perceived as likely to achieve its purpose (perceived effectiveness) and participants’ confidence to take and persist in the taking of PrEP (self-efficacy).

## Materials and methods

### Study design and setting

This was a qualitative methods study nested in an HIV vaccine preparedness study in Masaka district, southwestern Uganda. Between July 2018 and November 2019, 200 HIV negative men and women (18–45 years) determined to be at high risk of HIV acquisition were enrolled into the HIV vaccine preparedness study. Volunteers were recruited from places with known key populations and high HIV burden including trading towns along the trans-African highway [14–16] and fishing communities on the shores of Lake Victoria [17–21]. HIV risk was measured using an interviewer-administered risk assessment questionnaire. Individuals were considered to be at risk if they reported suspected/confirmed sexually transmitted infection (STI) or unprotected sex with two or more partners or unprotected sex with a new partner in the past three months or unprotected sex in exchange for money/goods in the past month. Participants were followed up every 3 months. At all visits, study participants were given information on oral PrEP including its benefits and possible side effects. A written referral to a PrEP provider was given to individuals who were willing to start PrEP and adherence counselling to those that initiated it.

### Study participants, sampling, and data collection

Ten percent [20] of the HIV vaccine preparedness study participants were purposively selected to participate in in-depth interviews (IDIs). Those selected included people of different ages, sex, occupational backgrounds, and residence. Willingness to take-up PrEP was not an inclusion criterion. Interviews were conducted using a topic guide at three and nine months after enrolment. Topics covered included individuals’ recent life history including work experience, relationship history and places lived, perceptions of HIV risk (including own risk), knowledge of and use of PrEP. The first interview was intended to establish rapport, to engage in a more personal and potentially sensitive conversation and to capture initial information. In addition to strengthening trust, the second (follow-up) interview filled gaps from the first interview as well as capturing new developments in the participant’s life including changes in work, relationships, residence, and HIV risk perceptions. It also sought more detail on issues around PrEP including any new information, such as change in attitudes, desire to take PrEP, and reasons for not taking up PrEP.

In addition, four focus group discussions (FGDs) were conducted among 29 participants who did not participate in the IDIs. The groups were defined as follows: younger (18–24 years) men, younger (18–24 years) women, older (30+ years) men and older (30+ years) women. Information on PrEP uptake was not considered in selecting individuals for FGDs. Each group comprised six to 10 participants. Issues discussed in each FGD included HIV risk, knowledge and uptake of PrEP, and reasons for non-uptake.

All IDIs and FGDs were conducted in locations that were convenient and safe for the participants and the researchers, and that ensured privacy. No audio recorders were used as these often obstruct discussion especially on sensitive topics [22]. A male and female interviewer conducted interviews, matched by sex with participants, and took short notes during each interview, which they expanded within 24 hours after the interview. Debriefing meetings with the interviewers and the lead social scientist were held after each interview to ensure completeness of the data. Any gaps identified were filled during subsequent interviews. All interviews were conducted in Luganda, the local language in the study area. On average, IDIs lasted for about one hour while FGDs lasted between one and a half to two hours.

### Data management and analysis

The research team conducted preliminary analysis of data collected in real time as scripts were prepared grouping information in topics and informed subsequent interviews [22]. Anonymized scripts from expanded notes were saved on password-protected computers. Data analysis was conducted by the interviewers, with support from another social scientist, and used a thematic framework approach guided by the TFA. Interview scripts were initially read independently by the two interviewers, to identify recurring significant themes relevant to the constructs of the TFA. We identified five themes that correlated with the constructs of the TFA, with some of the themes cutting across the constructs. The themes included: knowledge (or lack of) about PrEP for **intervention coherence**, attitudes to PrEP for **Affective attitude, Opportunity cost** and **Perceived effectiveness**, barriers for **Burden**, motivation to take PrEP for **Ethicality**, and persistence on PrEP for **Self efficacy**.

The identified themes were shared and discussed with the supporting social scientist and based on the seven components of the TFA, a coding framework was developed. Codes with similar or close meanings were grouped together to generate broader themes. Data were charted on a matrix under relevant/matching themes to provide an overview and enable quick and easy navigation.

### Ethical considerations

Ethical approval was obtained from the Uganda Virus Research Institute Research Ethics Committee (UVRI REC # GC/127/18/03/637) and from the Uganda National Council for Science and Technology (UNCST # HS 2392). Written informed consent to participate in all study procedures, including IDIs and FGDs was obtained at enrolment into the HIV vaccine preparedness study. Before each IDI and FGD, study staff verbally checked to confirm that participants were still interested in participating in this component of the study. Participants’ privacy and confidentiality were ensured at all stages of the study by conducting interviews in a safe and private place plus anonymizing and securely storing all collected data.

## Results

### Demographic characteristics of study participants

Half (10) of the IDI participants were women. The average age of the participants was 26 years and 29 years for men and women respectively. Six of the 10 men were married/cohabiting or in a relationship, compared to four out of the 10 women. Most participants had only primary school education (10) or no education (5). Half (5) of the men were involved in fishing while seven women either earned solely (3 women) or supplemented (4 women) their incomes with sex work. Among the FGD participants, 15 (52 percent) were men. Eleven (8 men and 6 women) participants were aged 18–24 years while the rest (7 men and 8 women) were ≥30 years (see Table 1).

**Table 1:** Characteristics of study participants.

Characteristic	IDI			FGD				
	Men	Women	All	Men (18–24 years)	Men (≥30 years)	Women (18–24 years)	Women (≥30 years)	All
**All**	10	10	**20**	8	7	6	8	**29**
**Mean age (years)**	26	29	**27.5**	23	36	23	34	**23 (18–24 years)**
								**35 (≥30 years)**
**Education**								
Primary	5	5	**10**	6	4	2	4	**16**
Secondary	3	2	**5**	2	1	3	3	**9**
No formal education	2	3	**5**	0	2	1	1	**4**
**Marital Status**								
Married	5	2	**7**	4	5	2	2	**13**
Single	4	3	**7**	1	2	2	4	**9**
Separated	0	3	**3**	1	0	0	1	**2**
In a relationship	1	2	**3**	2	0	2	1	**5**
**Occupation**								
Sex worker	0	3	**3**	0	0	0	2	**2**
Shopkeeper	0	2	**2**	0	0	0	0	**0**
Launderer	0	1	**1**	0	0	0	0	**0**
Food vendor	0	1	**1**	0	0	1	0	**1**
Fishing-related work	5	0	**5**	0	3	3	0	**6**
Mason	2	0	**2**	1	0	0	0	**1**
Mechanic	1	0	**1**	2	0	0	0	**2**
Electrician	1	0	**1**	0	0	0	0	**0**
Farmer	1	0	**1**	0	3	0	2	**5**
Salon/Bar/lodge worker	0	1	**1**	0	0	1	3	**4**
Motorcycle taxi rider	0	0	**0**	2	0	0	0	**2**
Disc jockey	0	0	**0**	1	0	0	0	**1**
Other	0	0	**0**	1	1	0	0	**2**
None	0	2	**2**	1	0	1	1	**3**

### Intervention coherence

In the TFA, this construct is defined as the extent to which participants understand the intervention and how it works. Participants described their ‘knowledge about PrEP’ including: meaning and uses of PrEP, who should take PrEP and PrEP dosing. Nevertheless, participants also had misconceptions regarding the use of PrEP.

At three months of participation in the cohort study, participants had some information regarding PrEP, although it was limited. Most participants knew that PrEP was a pill (in the same form as ARVs), taken daily by HIV uninfected individuals to prevent acquisition of HIV.

*“We were taught that PrEP prevents one from contracting HIV if taken like ARVs*, *that is*, *taken every day until one feels that he/she is no longer at risk of contracting HIV”* (man, 31 years).

Participants also knew that PrEP was meant for individuals with elevated risk of HIV such as those in HIV discordant relationships or with multiple sexual partners or that engage sex work, and that one could stop PrEP if they felt that their HIV risk was low. Indeed, a young man narrated how he stopped PrEP after his risk reduced:

“…*It happened that I was in a school holiday and had nowhere to access condoms yet I could land into temptations (of having sex)*. *When school resumed*, *the risk ended so I stopped PrEP”* (FGD, younger men).

Others explained how PrEP worked:

*“It (PrEP) is one of the HIV prevention measures*. *It could be used if you feel at risk of HIV or if you regularly get exposed to HIV; you can take PrEP to prevent you from catching HIV”* (FGD, younger men).

Participants however lacked full information regarding its effectiveness, proper dosage or where it could be accessed. Indeed, given that PrEP was still new to many, some had misconceptions that it was still undergoing clinical trials and could harm them. Others doubted whether it could really prevent one from acquiring HIV, *“I have tried to tell different people about PrEP but they strongly disagree that any drug can prevent HIV”* (man 24 years). While some, despite the fact that they had initiated taking it, did not believe in it either: “*I am just swallowing but I am not sure if it works*, *that is why I use other protective measures like condoms”* (woman, 30 years). Some individuals who were planning to initiate taking PrEP were also not convinced that it worked as expressed below:

*“I cannot rely on the fact that I am swallowing PrEP*, *I will continue with using condoms*. *I sleep with many different people (men) and I see the kind of fluids that they release; I cannot risk”* (woman, 30 years).

Information regarding PrEP was discussed with friends or family after learning about it at the research centre. However, in some cases, they would get negative or conflicting feedback, and this would distort their understanding of PrEP and thus discourage them from initiating or persisting in taking it. A woman who stopped using PrEP after experiencing side effects observed:

*“My friends advised me to drop the drug (PrEP) after taking for 15 days*. *They (friends) asked*, *‘what will you do when you catch HIV*?*’ Implying that I was taking ARVs when I was HIV negative yet it was meant for the HIV-infected and [it was not yet the] time for that”* (woman, 37 years).

### Affective attitude

This TFA construct describes how an individual feels about the intervention. In relation to the construct, we captured participants’ ‘attitudes to PrEP’, which included both perceived benefits and experiences of PrEP as well as their concerns.

Half of the participants acknowledged that PrEP was good especially for those in discordant or multiple sexual relationships. As one 23 year old man observed, this would allow the person on PrEP: *“to remain with the positive partner for the sake of raising the children together”*. Some who were practicing sex work were aware that PrEP would benefit them as expressed below:

*“Since I am exposed to many ways (partners) of contracting HIV*, *I was attracted because (PrEP) could protect me from contracting it (HIV)*. *For instance*, *if my sexual client is infected and I am not*, *I could remain protected from getting it”* (woman, 30 years)

The theme ‘attitudes to PrEP’ cut across different constructs of the TFA, including **perceived effectiveness**, which examines the extent to which an intervention is perceived as likely to achieve its purpose. A sub theme on usefulness of PrEP was identified related to this construct.

*“Some of us have like three women and you do not use condoms with them because you know they are your women*. *In case one of these women is cheating on you with another man*, *you are safe if you are using PrEP*. *Others are ‘safari’ (long distance) drivers and may travel away from home and wives*, *they may have sex with casual partners during their travel and in this case if you are taking PrEP*, *you have protection”* (FGD-men)

At the follow-up interview, over half of the interview participants (seven women and six men) expressed a desire to take PrEP, and four had initiated it, although one had discontinued:

*“… They gave me a bottle of pills (PrEP) for one month*, *but I swallowed for only two weeks and stopped due to side effects*. *I was experiencing dizziness*, *headache*, *general body weakness*, *hunger (increased appetite) and vomiting*. *I had thought it was just one pill to take me for three months and did not know that I was to take it daily*. *It causes a lot of thirst and if you do not take lots of fluids (drinks)*, *you are in trouble… I can never swallow it again*. *The truth is*, *if you do not have money*, *you should not swallow that drug (PrEP)”* (woman, 22 years).

However, while many felt PrEP would serve a good purpose in preventing HIV, participants had some concerns such as accessing PrEP from the same facilities as those which provide HIV treatment: *“I feel I cannot go to the other place (PrEP/ART facility)*, *I do not like the place”* (woman, 36 years) and also the reaction of persons that are close to them if they found out that they were taking PrEP as illustrated here:

*“A friend of mine who is HIV positive told me it (PrEP) was like the drugs she was getting*. *I even felt ashamed because I had told her that the health care providers had tested me for HIV*, *and they gave me a negative test result*. *But then here I was*, *taking similar drugs*. *It would appear like I was bragging that I was HIV negative*, *yet the friend is HIV-infected and this claiming that I was HIV negative would make her feel bad”*. (woman, 35 years).

With these mixed feelings of liking the idea of PrEP and also fearing for PrEP, many decided to wait or postpone initiating it as they observed what happened to those who were taking it. Others just felt it did not make sense to take drugs when one is healthy as expressed by a 30-year-old woman responding to questions about her non-uptake of PrEP:

*“People fear it (PrEP). For example, a friend of mine feared to take it and said she could not start drugs before she gets infected. ‘What if it causes me problems?’ This friend asked”*.

### Burden

According to the TFA, burden is the perceived amount of effort required to participate in the intervention. We identified barriers to uptake of/and persistence on PrEP as themes under this construct.

The barriers were both perceived and experienced, which included both monetary and non-monetary costs. These ranged from the long distances to PrEP facilities, burden of daily pill taking, drug side effects, and also fear of stigma. For instance, the long distances to PrEP facilities resulting in high transport costs were an impediment to uptake or adherence to and persistence on PrEP in the case of some who failed to go for re-fills. One participant described how she had to share PrEP with a friend because she could not afford transport to go and pick up her own.

*’’Personally*, *I did not have money to go to xxxx where they had referred me*, *so I first shared PrEP with a friend who had it in plenty*. *She gave me pills for like three months and after taking it and it worked for me (gave me good health) because I even stopped getting infections like flu or cough*, *I decided to go and get my own drugs”* (FGD-women).

Besides the general dislike for pills, the perceived burden of taking a daily pill with the similar packaging to ARVs, caused individuals to shy away from PrEP for fear of being labelled as HIV-infected.

*“If PrEP could be packed like this (pulls out a pill sachet)*, *people would not suspect it to be HIV drugs and they would carry it comfortably*. *Unlike as it is now whereby they are in these plastic containers and make a lot of noise as you move*. *If it were sealed in a sachet*, *I would not have had any problem carrying it or pulling it out and swallow the drug in the presence of other people as that packaging looks like any normal drugs”* (woman, 23 years).

Some participants reported discontinuing PrEP due to side effects like nausea, headaches, hallucinations, dizziness and general body weakness among other side effects, for others it was the fear of these expected problems which barred them from initiating PrEP.

*“… people may not take PrEP thinking that it might have many side effects*. *For example*, *causing headache and general body weakness given the nature of our work (fishing)*, *which needs much energy*. *Weakening the body and making it prone to diseases like HIV”* (man, 31 years)

### Ethicality

This TFA construct is about the extent to which the intervention fits well with an individual’s value system. We identified the theme of Motivation to take PrEP as being related to this construct, whereby perceived HIV risk motivated participants to take a positive step to protect their health by taking PrEP.

Many participants, especially the younger ones, felt that PrEP was the ideal HIV prevention choice for them, given that almost all of them did not like or rarely used condoms. Even the women, especially those involved in sex work acknowledged their HIV-risk and felt PrEP would greatly help them as their sexual clients sometimes forced them into unprotected sex, intentionally broke condoms or even offered and paid higher prices for unprotected sex. Even the non sex workers did not trust their regular partners and felt PrEP would give them protection in case the partners contracted HIV from elsewhere.

*“I am still at the sexually active age*, *so*, *that method (PrEP) will be very beneficial to me*. *A man may refuse to use a condom or may use it but then remove it during the process and for that*, *I cannot rely on condoms alone”* (woman, 40 years).

### Opportunity cost

Defined in the TFA as the extent to which benefits, profits or values must be given up in order to engage in an intervention we found this was linked to the cross cutting theme of attitudes to PrEP. Our analysis revealed participants’ concerns around unintended disclosure that came with taking PrEP.

Some participants felt that taking PrEP meant losing one’s privacy, trust and also respectability. They noted that it was hard to take PrEP without others noticing and as much as one may hide, others would eventually get to know. This woman (30 years) said, sadly, that:

*“I disclosed to my sister about my taking PrEP after she saw me taking the pills*, *but the sister instead told our mother that I am HIV positive and on treatment*. *My sister’s child also supplemented that she saw me taking the pills from the toilet*, *which was not true”*.

Men expressed similar fears, a 24 year old man said:

*“Some other people fear to be known that they are taking PrEP by their friends and relatives/family because they will judge them and sometimes suspect them to have HIV”*.

Participants felt that such disclosure of PrEP use, whether intentional or not could be misinterpreted and expose one’s infidelity or promiscuity and could result in suspicion, distrust or loss of partner, as well as domestic violence in some extreme cases. Indeed, in the FGDs conducted with men, they preferred not to disclose their use of PrEP to their more stable partners because they felt it would jeopardize those relationships

*“No*, *it would be better if my wife did not know about my taking PrEP because she will think I am womanising since we always test negative together”* (FGD-men).

### Self-efficacy

Defined using the TFA as participants’ confidence to take and adhere to PrEP. The construct was linked to the theme on persistence on PrEP.

As we have noted above, the awareness that PrEP would protect them from acquiring HIV motivated some of the participants to start using it despite the perceived/experienced challenges associated with taking PrEP, *“Everyone loves his/her life and people will surely take and adhere to PrEP if they are sure that they are protected from HIV”* (man 44 years). They reported devising ways to facilitate their PrEP taking and adherence, so they could persist with regular PrEP use. For example, one of the women initially had problems swallowing the pills due to the big size, she recalled how she finally, comfortably, took her PrEP:

*“Even if I do not have money for transport to go for refills*, *I can walk to the PrEP facility because is it near […] however when I had just started PrEP*, *I found the pill big to swallow but I would break it into two pieces and still swallow*. *I am now used to the pill and no longer have issues with the size*. *I disclosed to household members including my partner that I am on PrEP and given the fact that I disclosed*, *I freely take my PrEP without hiding the pills nor taking them stealthily*. *Not even the girls (partner’s granddaughters) hide their pills (PrEP)*. *We keep our PrEP in the open and if you visit our home and see it*, *it is all up to you”* (woman, 40 years).

Generally, many participants expressed a liking for PrEP and acknowledged that it would benefit them particularly because of their risk of HIV infection. However, this did not always translate into uptake, as we outline above, as many had concerns about the nature and dosing of pills as well as the structures through which those pills are being delivered.

## Discussion

Our study findings show that individuals at high risk of HIV infection who were enrolled in an HIV vaccine preparedness study in Uganda were interested in taking oral PrEP because they understood that it would protect them against acquisition of HIV, a finding corroborated by other recent work in Uganda [11, 23, 24]. However, among those who had chosen to start PrEP many did not initiate and/or persist with the drug. This was attributed to the burden of daily oral pill taking, concerns regarding side effects, and stigma. To some extent, the negative influence from other people as well as the lingering doubts regarding the drug’s efficacy negatively influenced uptake. In addition, the limited understanding of PrEP as well as the fears/concerns exhibited by our study participants prevented some of them from taking up the intervention much as they considered it beneficial, as documented in other studies [25]. Research has shown how anticipation of negative effects can influence intervention acceptance, uptake, and adherence [23, 26, 27].

To build individuals’ confidence in their ability to take PrEP [self-efficacy], it is necessary that they get detailed information on how PrEP works, what to expect in terms of side effects, and how to deal with these issues [28–30]. The common reports of stigma hindering uptake of PrEP among our study participants were similar to the findings from other studies [11, 31]. For example, research on uptake of PrEP has reported that stigma associated with being labelled as HIV-infected and being suspected of infidelity or promiscuity, was a barrier to uptake of PrEP [11, 32, 33].

Similarly, the stigma related to the PrEP facility which was the same place where people on ARVs picked their refills was noted. As a result, participants in this study expressed their dislike for the PrEP/ARV facility and said they could not go there to pick PrEP. This was in agreement with earlier research [34], which found that the integration of PrEP with HIV care services hindered PrEP uptake, thus emphasising the need for alternative PrEP delivery approaches. PrEP needs to be integrated into broader health promotion and divested of its association with HIV-treatment structures using differentiated service delivery through community-based structures. Such changes to delivery would be influential in terms of improving access and acceptability.

We found that some participants who initiated PrEP stopped taking it after experiencing side effects. This reflects that acceptance of an intervention may change as people gain experience with using it as well as more information and understanding about the side effects themselves. This change may sometimes be related to the burden that is associated with taking on the intervention [26, 35]. Whereas previous conceptualization of acceptability emphasizes that individual perceptions of an intervention greatly influences its acceptability [12], our findings concur with Sekhon’s argument that acceptability is dynamic and may change after experiencing the intervention [13].

Bandura [36] suggests that the amount of effort that people invest in taking up an intervention will translate to uptake and the level of resilience when faced with challenges: ‘The stronger the perceived self-efficacy, the more active the efforts’ (p. 194) and ‘The stronger the efficacy expectation, the higher was the likelihood that a particular task would be successfully completed’ (p. 207). Indeed, participants in our study who initiated PrEP but seemed not to believe in its efficacy, were likely not to persist with it. Research has shown that an individual may reinforce their self-efficacy if they believe the intervention will benefit them [37] and they receive support from research/health care staff. For example, providing individuals with risk information and reminders of their risk behaviour can change their assessments of risk [38, 39]. Therefore, interventions to address such concerns may improve acceptability.

The belief held by a few of our participants that they did not need to take PrEP because they were not at risk or had other HIV prevention methods, corroborates the findings of other studies which show that individuals exhibiting optimal beliefs in susceptibility are not likely to accept any recommended health action [25, 40]. Therefore, HIV prevention interventions need to be explicit as to what might cause HIV risk if these interventions are to benefit the targeted groups of key or high risk populations [41]. Again, some individuals are reluctant to disrupt or abandon other HIV prevention methods like condoms or couple HIV counselling and testing which they believe to have worked well for them and which they highly value [26].

A limitation of our study was that our participants were selected from a vaccine preparedness study cohort where they regularly received health education and HIV prevention services including information on PrEP. However, the latter could have been affected by staff perceptions and attitudes towards oral PrEP. Hence, our findings may not be generalizable to other at-risk populations who do not get such services. Further still, the association of PrEP with stigmatised populations, experiences and attitudes on PrEP may differ in a general population where there is less ‘branding’ or targeting of certain groups [34]. Nevertheless, our study provides useful insights into the challenges of PrEP use among individuals at high risk of HIV infection in the study area and similar settings. The combination of repeat IDIs and FGD data allowed for a deeper understanding of participants’ knowledge and acceptability of PrEP.

## Conclusion

We observed considerable interest in taking PrEP among our study participants. However, limited understanding of and concerns about PrEP could impede its uptake and sustained use. Provision of comprehensive information about PrEP, instituting measures to destigmatize PrEP use by integrating into broader health promotion and providing in non-health facility settings, plus the introduction of more user-friendly PrEP formulations, such as injectable PrEP, could improve uptake and adherence.

## Supporting information

S1 File Registration cohort–baseline data. (XLSX)

S2 File Registration cohort–followup data. (XLSX)
